# Development of cardiometabolic risk factors following endocrine therapy in women with breast cancer

**DOI:** 10.21203/rs.3.rs-2675372/v1

**Published:** 2023-03-22

**Authors:** Eileen Rillamas-Sun, Marilyn L. Kwan, California Carlos, Richard Cheng, Romain Neugebauer, Jamal S. Rana, Mai Nguyen-Huynh, Zaixing Shi, Cecile A. Laurent, Valerie S. Lee, Janise M. Roh, Yuhan Huang, Hanjie Shen, Dawn L. Hershman, Lawrence H. Kushi, Heather Greenlee

**Affiliations:** Fred Hutchinson Cancer Center; Kaiser Permanente Northern; Iribarren Kaiser Permanente Northern California; University of Washington School of Medicine; Kaiser Permanente Northern California; Kaiser Permanente Northern California; Kaiser Permanente Northern California; Xiamen University School of Public Health; Kaiser Permanente Northern California; Permanente Northern California; Kaiser Permanente Northern California; Fred Hutchinson Cancer Center; Fred Hutchinson Cancer Center; Columbia University Irving Medical Center; Kaiser Permanente Northern California; Columbia University

**Keywords:** endocrine therapy, tamoxifen, aromatase inhibitors, cancer survivors, cardiometabolic risk, cardiovascular disease

## Abstract

**Purpose:**

Studies comparing the effect of aromatase inhibitor (AI) and tamoxifen use on cardiovascular disease (CVD) risk factors in hormone-receptor positive breast cancer (BC) survivors report conicting results. We examined associations of endocrine therapy use with incident diabetes, dyslipidemia, and hypertension.

**Methods:**

The Pathways Heart Study examines cancer treatment exposures with CVD-related outcomes in Kaiser Permanente Northern California members with BC. Electronic health records provided sociodemographic and health characteristics, BC treatment, and CVD risk factor data. Hazard ratios (HR) and 95% con dence intervals (CI) of incident diabetes, dyslipidemia, and hypertension in hormone-receptor positive BC survivors using AIs or tamoxifen compared with survivors not using endocrine therapy were estimated using Cox proportional hazards regression models adjusted for known confounders.

**Results:**

In 8,985 BC survivors, mean baseline age and follow-up time was 63.3 and 7.8 years, respectively; 83.6% were postmenopausal. By treatment, 77.0% used AIs, 19.6% used tamoxifen, and 16.0% used neither. Postmenopausal women who used tamoxifen had an increased rate (HR: 1.43, 95% CI: 1.06–1.92) of developing hypertension relative to those who did not use endocrine therapy. Tamoxifen use was not associated with incident diabetes, dyslipidemia, or hypertension in premenopausal BC survivors. Postmenopausal AI users had higher hazard rates of developing diabetes (HR: 1.37, 95% CI: 1.05–1.80), dyslipidemia (HR: 1.58, 95% CI: 1.29–1.92) and hypertension (HR: 1.50, 95% CI: 1.24–1.82) compared with non-endocrine therapy users.

**Conclusion:**

Hormone-receptor positive BC survivors treated with AIs may have higher rates of developing diabetes, dyslipidemia, and hypertension over an average 7.8 years post-diagnosis.

## Introduction

An estimated 83% of all breast cancers (BC) are hormone receptor-positive [1], and endocrine therapy is typically the primary adjuvant treatment for these cancers. Endocrine therapies have been effective at treating hormone receptor-positive BC with five-year survival rates ranging from 89–92% [1]. Studies have shown that the long-term cardiovascular impact of endocrine therapy is an important health concern for BC survivors and an active area of research [2–4].

The most used endocrine therapies are selective estrogen-receptor modulators (i.e., tamoxifen) and aromatase inhibitors (AIs), which have different modes of action in treating hormone receptor-positive BC. Tamoxifen blocks estrogen from binding to receptors on breast cancer cells, but mimics estrogen in other tissue cells [1, 4]. Tamoxifen is a treatment used in both premenopausal and postmenopausal women. Via a different mechanism, AIs reduce endogenous estrogen production by blocking aromatase, which is the enzyme that converts androgens to estrogens in non-ovarian tissues [4]. Since the ovaries produce the majority of endogenous estrogen, AIs are primarily used in postmenopausal women whose ovarian function has ceased. AI use has been associated with lower breast cancer recurrence and mortality compared with tamoxifen [5, 6], but the impact of AIs on cardiovascular health should be considered in the adjuvant setting [7]. Studies have been published on the cardiovascular disease (CVD) risks associated with endocrine therapies [6, 8–15], including several meta-analyses and systematic reviews [3, 16, 17]. Observational studies often directly compare AI and tamoxifen users, however endogenous estrogen, which premenopausal women have in higher concentrations, is generally associated with favorable cardiometabolic factors, such as reduction in cardiomyocyte hypertrophy, inammation, and atherosclerosis [2, 4, 18] and improvements in lipid pro les and insulin resistance [18, 19]. Since AIs work by suppressing endogenous estrogen, AIs may more negatively affect the cardiovascular system than tamoxifen. Thus, opposing effects on the cardiovascular system between the two therapies challenge the capability to distinguish whether associations between endocrine therapy use and CVD are due to a protective effect of tamoxifen or a deleterious effect of AIs. Clearer results might emerge by comparing AI or tamoxifen users to hormone-receptor positive breast cancer survivors who did not use endocrine therapy for treatment.

Therefore, we examined the association of AI or tamoxifen use on the risk of developing diabetes, dyslipidemia, and hypertension in a large population of hormone-receptor positive BC survivors. To minimize confounding and improve clarity in potential associations, AI-only and tamoxifen-only users were separately compared with BC survivors who had an indication, but did not use any endocrine therapy for cancer treatment. Finally, since higher body mass index (BMI) is a risk factor for diabetes, dyslipidemia, and hypertension, we explored whether these relationships varied by baseline BMI category.

## Methods

### Study Population

The Pathways Heart Study is a prospective cohort study within Kaiser Permanente Northern California (KPNC) whose aim is improve understanding of CVD risks and outcomes associated with cancer treatments in women with a history of BC. Women were eligible for participation in the Pathways Heart Study if they were diagnosed with American Joint Committee on Cancer (AJCC) Stage I-IV invasive BC between November 2005 and March 2013, at least 21 years of age, and had active KPNC membership for one year or longer at the time of their BC diagnosis. Eligible study participants for the Pathways Heart Study were identified from KPNC electronic health records (EHR). For this analysis, participants who did not have hormone-receptor positive BC, were diagnosed at AJCC Stage IV, and/or were underweight (defined as BMI < 18.5 kg/m^2^) were excluded. Furthermore, women with a history of using both therapies were excluded, given the exposure of interest was history using AI only or tamoxifen only for cancer treatment. Thus, the comparison group was hormone-receptor positive BC survivors with no history taking AI or tamoxifen for their BC treatment.

### Measures

Data were obtained from the KPNC EHR and included KPNC membership enrollment, sociodemographic characteristics, select clinical measures, and incident and prevalent diabetes, dyslipidemia, and hypertension. Menopausal status at baseline (defined as date of BC diagnosis) was not available in the EHR for all women, so a baseline age cutoff of 51 years or older was used to characterize postmenopausal status [20]. BMI was calculated as weight, in kilograms, divided by height, in meters, squared. BMI values were grouped into standardized categories used to characterize people with normal weight, overweight, and obesity [21]. Use of AI or tamoxifen endocrine therapy use was identified KPNC outpatient pharmacy data.

Characterization of incident diabetes, dyslipidemia, and hypertension using KPNC EHR data has been described in detail [22]. Incident diabetes was identified the KPNC Diabetes Registry [23] based on one or more ICD-9/10-CM principal inpatient diagnosis codes, two or more outpatient diagnoses in the previous five years, two or more fasting blood glucose lab result of ≥ 126 mg/dL on separate days over the last two years, or one or more prescribed diabetes medication. Women with incident dyslipidemia had two separate diagnosis codes of ICD-9-CM 272.0–272.4, ICD-10 E78.00, E78.01, E78.1-R78.5 or a combination of two or more of the following: one diagnosis code as above, a low-density lipoprotein cholesterol result of ≥ 160 mg/dL, or a dispensed lipid-lowering medication such as a statin or other antilipemic agent. Incident hypertension was defined as either: 1) having two or more hypertension diagnoses of ICD-9-CM 401.xx (or equivalent ICD-10-CM codes) at primary care visits in the previous two years, or 2) one or more primary care hypertension diagnoses and either one or more hospitalization with a primary or secondary hypertension diagnosis in the previous two years, or 3) one or more dispensed hypertension medication in the previous 6 months [24]. If two diagnosis codes were required to identify incidence of the cardiometabolic risk factor, the earliest diagnosis date was considered the diagnosis date.

### Statistical Analysis

Baseline demographic and health characteristics of the study population by AI or tamoxifen use and menopausal status were described using means and standard deviations (SD) and frequencies. Women with history of AI or tamoxifen use were compared with hormone-receptor positive BC survivors who did not use any endocrine therapy for their BC treatment. Since tamoxifen is indicated for both premenopausal and postmenopausal women, analyses stratified tamoxifen users by menopausal status. Analysis for AI users were in postmenopausal women only. Outcomes of interest were incident diabetes, dyslipidemia, and hypertension; women with a history of these conditions at baseline were excluded from the analysis of that specific risk factor.

Cox proportional hazards regression models were used to estimate the hazard rate ratios (HR) and 95% confidence intervals (CI) of newly developed diabetes, dyslipidemia, and hypertension in AI or tamoxifen users compared with hormone-receptor positive non-users of endocrine therapy. Models adjusted for race/ethnicity, baseline age, smoking status, BMI, cancer stage, history of chemotherapy, history of radiation therapy, and prevalent diabetes, dyslipidemia, and hypertension (when not the incident outcome).

To evaluate the HR and 95% CI of developing diabetes, dyslipidemia, and hypertension within each of the three BMI groups, Cox proportional hazards regression models were stratified by participants with normal weight, overweight, and obesity. To determine whether the HRs were statistically different between the BMI groups, we evaluated Cox proportional hazards regression models that included two multiplicative interaction terms, one for endocrine therapy use (AI or tamoxifen) by overweight and one for endocrine therapy use (AI or tamoxifen) by obesity. These interaction terms compared whether the HR for AI (or tamoxifen) users with overweight (or obesity) relative to AI (or tamoxifen) users with normal weight were statistically different, which would suggest that the associations of these endocrine therapies on the development of these cardiometabolic factors varied by baseline BMI category. P-values ≤ 0.05 were considered statistically significant and analyses were completed using SAS version 9.4 (SAS Institute Inc., Cary, NC).

## Results

The KPNC EHR identified8,985 hormone-receptor positive BC survivors eligible for analysis, of whom 16.4% (n = 1,472) were premenopausal and 83.6% (n = 7,513) were postmenopausal. Among premenopausal BC survivors, 82.3% (n = 1,212) used tamoxifen. Among postmenopausal survivors, 7.3% (n = 546) used tamoxifen, while 77.0% (n = 5,788) used AIs. Participants in our sample were followed over mean (SD) 7.8 (3.8) years. Table 1 describes the baseline characteristics of the study population by menopausal status and endocrine therapy use.

For premenopausal women with hormone-receptor positive BC, tamoxifen and non-users of endocrine therapy were similar in neighborhood education and income, BMI, smoking status, and prevalence of diabetes, dyslipidemia, and hypertension. However, tamoxifen users were slightly younger (mean age 44.0 vs. 46.2 years, p < 0.001), more likely to be Asian or Pacific Islander (24.7% vs. 15.4%, p = 0.001), AJCC Stage II or III (46.9% vs. 29.3%, p < 0.001), and have received chemotherapy (57.2% vs. 31.5%, p < 0.001).

Among postmenopausal hormone-receptor positive BC survivors, tamoxifen, AI, and non-users of endocrine therapy had different baseline characteristics (Table 1). Compared with non-endocrine users, postmenopausal tamoxifen users had similar racial/ethnic distributions, neighborhood incomes, and BMI levels. However, tamoxifen users were younger (mean age 66.6 vs. 71.1 years, p < 0.001), diagnosed at AJCC Stages II-III (30.6% vs. 23.1%, p = 0.001), never smokers (57.9% vs. 51.3%, p = 0.04), have a history of chemotherapy (18.5% vs. 9.5%, p < 0.001) and radiation (61.4% vs. 49.4%, p < 0.001), and were less likely to have diabetes (13.0% vs. 18.5%, p = 0.005), dyslipidemia (46.2% vs. 52.2%, p = 0.02), and hypertension (47.8% vs. 57.0%, p < 0.001). In contrast, postmenopausal AI users were similar in neighborhood education levels, smoking status, and prevalence of diabetes, dyslipidemia, and hypertension relative to non-endocrine users, but were more likely to be Asian or Pacific Islander (13.1% vs. 9.8%, p < 0.001), AJCC Stage II (32.8% vs. 18.3%, p < 0.001), with obesity (40.1% vs. 30.5%, p < 0.001), and have received chemotherapy (29.9% vs. 9.5%, p < 0.001) and radiation therapy (68.9% vs. 49.4%, p < 0.001).

The hazard rate ratios of incident diabetes, dyslipidemia, and hypertension by baseline menopausal status for tamoxifen or AI users relative to non-users of endocrine therapies are shown in Table 2. Tamoxifen use was not associated with developing diabetes or dyslipidemia in either premenopausal or postmenopausal BC survivors, even after adjusting for known confounders. However, postmenopausal tamoxifen users had a HR of 1.43 (95% CI: 1.06, 1.92) of developing hypertension relative to postmenopausal non-endocrine users, after adjustment. AI use was associated with higher HR of incident diabetes, dyslipidemia, and hypertension in unadjusted models, and these hazard rates remained statistically significant after adjustment (Table 2). The HR of diabetes attenuated from 1.74 (95% CI: 1.34, 2.25) in unadjusted models to 1.37 (95% CI: 1.05, 1.80) after adjusting for confounders. For dyslipidemia, adjustment also slightly attenuated the unadjusted HR of 1.62 (95% CI: 1.34, 1.96), but remained high at 1.58 (95% CI: 1.29, 1.92). However, the 1.50 (95% CI: 1.24, 1.82) adjusted HR of hypertension was an increase from 1.39 (95% CI: 1.16, 1.67) in unadjusted models.

The association of developing diabetes, dyslipidemia, and hypertension by BMI category in AI or tamoxifen users compared with non-endocrine users is shown in Table 3. The HR of developing diabetes, dyslipidemia, or hypertension in premenopausal tamoxifen users relative to premenopausal non-users of endocrine therapy and in postmenopausal AI users compared with postmenopausal non-users of endocrine therapy was not different between the BMI groups. Similarly, the HR of incident dyslipidemia and hypertension by BMI group were similar for postmenopausal tamoxifen users relative to postmenopausal non-users of endocrine therapy (Table 3). However, postmenopausal tamoxifen users with obesity had a HR of 2.05 (95% CI 1.13, 3.74) for diabetes relative to postmenopausal non-users of endocrine therapy with obesity. This HR was close to statistically different to the HR of 0.76 (95% CI: 0.26, 2.20) for incident diabetes found in postmenopausal tamoxifen users with normal weight relative to postmenopausal non-users of endocrine therapy with normal weight (p-value for interaction = 0.08).

## Discussion

This analysis of endocrine therapy use in 8,985 hormone-receptor positive BC survivors followed over an average 7.8 years showed that AI use was associated with higher rates of incident diabetes, dyslipidemia, and hypertension in postmenopausal women, and that these rates did not vary by BMI group at baseline. Tamoxifen use was not associated with developing dyslipidemia in either premenopausal or postmenopausal women or by BMI group at baseline. However, postmenopausal tamoxifen users may have higher hypertension rates and postmenopausal tamoxifen users with obesity might have higher rates of incident diabetes than tamoxifen users with normal weight.

We reported an increased risk of developing diabetes among postmenopausal AI users, but no association was detected among premenopausal tamoxifen users compared with hormone-receptor positive BC survivors with no history of endocrine therapy use for BC treatment. Further, we found a possible higher diabetes risk in postmenopausal tamoxifen users with obesity when compared with the diabetes risk in postmenopausal tamoxifen users with normal weight. Previously published studies on these relationships are inconsistent. In a study of 133,171 BC survivors identified the National Health Insurance Service database of Korea, incident diabetes was associated with a statistically significant hazard ratio of 1.24 for postmenopausal AI users and of 1.24 and 1.26 for premenopausal and postmenopausal tamoxifen users, respectively, relative to non-users [25]. A similar investigation using 22,257 BC survivors from the Taiwanese National Health Insurance Research Database reported a 1.32 rate of diabetes in tamoxifen survivors compared with non-endocrine therapy users, but a protective association (HR = 0.68, 95% CI: 0.60–0.78) with incident diabetes in AI users [26]. A meta-analysis of seven observational studies described a pooled adjusted diabetes risk of 1.30 (95% CI: 1.20, 1.40) for tamoxifen users and no association for AI users compared with non-users [27]. Prior publications have also reported no association of diabetes risk in tamoxifen users when compared with non-users [28] or AI users [29], while another study reported no relationship with incident diabetes in either tamoxifen or AI users when compared with healthy non-BC controls [30]. Like many of these previous studies, we also compared endocrine therapy users with BC survivors who had no history of endocrine therapy use for treatment, but we limited our sample to only those with hormone-receptor positive BC and it is unclear whether only hormone-receptor positive cases were examined in these other studies. Prior studies also did not appear to stratify by menopausal status or BMI groups, so impacts of aging ovarian function and overweight or obesity make comparisons with our analyses challenging.

AI only, but not tamoxifen only, users in our analysis were at increased rate of developing dyslipidemia. Our nding of no association with dyslipidemia in pre- and postmenopausal tamoxifen users is generally consistent with the existing literature. Studies suggest favorable lipid pro les, including decreased total cholesterol and low-density lipoprotein cholesterol and increased high-density lipoprotein cholesterol, for tamoxifen users in both premenopausal and postmenopausal BC survivors [31–42]. In contrast, prior research frequently reports no significant changes in lipid pro les with AI use [19, 34, 35, 41, 43, 44]. Furthermore, studies investigating dyslipidemia as an outcome endpoint reported no association in BC survivors comparing AI to tamoxifen users [40, 43]. Since tamoxifen use is associated with improvements in lipid biomarkers, its cardioprotective effects may mask any possible deleterious impacts of AI use when compared with each other. It is possible that our use of women with a history of hormone-receptor positive BC who did not use endocrine therapy as a comparison group may provide more clarity about these relationships and contribute to an explanation of our divergent findings. We recommend re-examining this analysis with these same comparators in another large sample of hormone-receptor positive BC women.

We also found that postmenopausal tamoxifen and AI users both had higher rates of developing hypertension relative to their postmenopausal non-endocrine therapy user counterparts. Although no variations were observed by BMI group at baseline, postmenopausal AI users with normal weight, overweight, and obesity at baseline all had similar and statistically significant elevated risks of incident hypertension, suggesting robustness in these findings and minimal impact of BMI on these associations. Fewer studies have been published on hypertension risk and endocrine therapy use in BC survivors. A cross-sectional study by Blaes *et al*. reported higher mean systolic blood pressure in 34 women with hormone-receptor positive BC survivors taking AIs compared with 25 postmenopausal women without BC [45]. However, a meta-analysis using 10 clinical trials by Boszkiewicz *et al*. found no association of AI use with systemic hypertension when compared with tamoxifen only users or non-users [43]. Similarly, studies have reported no relationship between tamoxifen use and higher systolic or diastolic blood pressures in postmenopausal women [38, 46]. These studies had follow-up times of two years or less and therefore, longer-term associations of AI and tamoxifen use on blood pressure could not be determined. Longer follow-up of endocrine therapy users may result in positive associations with incident hypertension. More studies are needed to con rm our findings.

This study has limitations. We did not consider specific details of endocrine therapy, such as dose, duration of use, length of time since treatment concluded during the follow-up period, and specific types of AIs (e.g., steroidal vs. non-steroidal), which might have introduced bias if large variations of these details occurred within our study sample. We excluded women with history of using both tamoxifen and AI, although we acknowledge that sequential use of these endocrine therapies for treatment of hormone-receptor positive BC is common clinical practice. Whether these same results would be observed in BC survivors who took tamoxifen while premenopausal and then switched to AIs after menopause is not known. We also examined cardiometabolic risk factors as binary outcomes and did not include specific, physiological biomarker levels, such as total cholesterol, lipoproteins, blood glucose and insulin, and systolic and diastolic blood pressures. Finally, it is unclear why the hormone-receptor positive BC survivors in our comparison group did not use endocrine therapy for their treatment, despite it being the standard of care for this type of BC. In general, women in the comparison groups were older, diagnosed with lower stages of BC, and less likely to have any history of BC treatment, including radiation, chemotherapy, and surgery. Although we adjusted for these variables in our statistical models, there may still be unmeasured confounding. A future study using the Pathways Heart Study data to improve our understand of the reasons why women do not undergo treatment is in development.

Strengths of the study included a large sample size of hormone-receptor positive BC survivors with ample statistical power to examine tamoxifen-only and AI-only associations and relationships by baseline menopausal status and BMI groups. We conducted analyses separately for tamoxifen users and AI users and used a comparison group of non-endocrine therapy hormone-receptor positive BC survivors, resulting in clearer associations on the impacts of these therapies on cardiometabolic risk. Studies comparing tamoxifen and AI users were likely confounded due to the potential cardioprotective bene ts of tamoxifen use, thus challenging the ability to identify potential associations of cardiometabolic risk with AI use [4].

In conclusion, postmenopausal hormone-receptor positive BC survivors treated with AIs may have increased risk of incident diabetes, dyslipidemia, or hypertension over an average 7.8 years of follow-up, while postmenopausal tamoxifen users may have increased risk of developing hypertension. It is unknown whether these associations in cardiometabolic risk factors translates to increased risk of future cardiovascular and cerebrovascular disease or poorer long-term cardiovascular health in these women. Further exploration and con rmation in similar study populations is recommended.

## Figures and Tables

**Table 1:** Baseline characteristics of women with hormone-receptor positive breast cancer by menopausal status and endocrine therapy use

	Premenopausal (n = 1,472)			Postmenopausal (n = 7,513)				
	Non-Users of Endocrine Therapy	Tamoxifen Users	p-value^1^	Non-Users of Endocrine Therapy	Tamoxifen Users	p-value	Aromatase Inhibitor Users	p-value^1^
N (%)	260 (17.7)	1,212 (82.3)		1,179 (15.7)	546 (7.3)		5,788 (77.0)	
Follow-up time, years, Mean (SD)	9.2 (2.8)	8.8 (2.7)	0.05	7.8 (4.0)	8.1 (3.6)	0.17	7.5 (4.0)	0.03
Age, years, Mean (SD)	46.2 (4.7)	44.0 (5.5)	< 0.001	71.1 (11.2)	66.6 (11.0)	< 0.001	66.2 (8.9)	< 0.001
Race/ethnicity, n (%)			0.001			0.69		< 0.001
White	162 (62.3)	586 (48.4)		862 (73.1)	392 (71.8)		4054 (70.0)	
Black	20 (7.7)	109 (9.0)		74 (6.3)	31 (5.7)		338 (5.8)	
Asian or Pacific Islander	40 (15.4)	299 (24.7)		116 (9.8)	61 (11.2)		759 (13.1)	
Hispanic	37 (14.2)	208 (17.2)		110 (9.3)	57 (10.4)		603 (10.4)	
American Indian or Alaskan Native	1 (0.4)	10 (0.8)		17 (1.4)	5 (0.9)		34 (0.6)	
Neighborhood median household income, Mean (SD)	$78,848 ($33,113)	$80,877 ($34,127)	0.38	$77,083 ($33,137)	$76,859 ($33,305)	0.9	$81,742 ($34,252)	< 0.001
Percent neighborhood education, Mean (SD)								
Less than high school	12.2 (10.7)	12.5 (11.6)	0.67	12.0 (11.5)	12.3 (11.2)	0.59	11.7 (11.1)	0.41
High school or GED	19.2 (10.7)	19.8 (10.0)	0.38	20.1 (10.3)	20.6 (9.7)	0.37	19.8 (10.1)	0.41
Some college	31.4 (10.1)	30.6 (10.3)	0.24	30.6 (10.5)	32.0 (10.4)	0.01	30.5 (10.2)	0.69
College graduate or more	37.2 (20.6)	37.1 (20.3)	0.94	37.2 (21.3)	35.1 (20.0)	0.04	37.9 (20.6)	0.29
AJCC Stage, n (%)			< 0.001			0.001		< 0.001
Stage I	184 (70.8)	643 (53.1)		907 (76.9)	379 (69.4)		3376 (58.3)	
Stage II	62 (23.9)	473 (39.0)		216 (18.3)	142 (26.0)		1900 (32.8)	
Stage III	14 (5.4)	96 (7.9)		56 (4.8)	25 (4.6)		512 (8.9)	
Received chemotherapy, n (%)	81 (31.5)	690 (57.2)	< 0.001	111 (9.5)	101 (18.5)	< 0.001	1722 (29.9)	< 0.001
Received radiation, n (%)	135 (51.9)	794 (65.5)	< 0.001	582 (49.4)	335 (61.4)	< 0.001	3985 (68.9)	< 0.001
Had breast surgery, n (%)	247 (95.0)	1188 (98.0)	0.005	1121 (95.1)	541 (99.1)	< 0.001	5675 (98.1)	< 0.001
Body mass index, kg/m^2^, Mean (SD)	27.5 (6.9)	27.5 (6.6)	0.93	28.0 (6.3)	27.4 (5.6)	0.045	29.4 (6.4)	< 0.001
Body mass index category, n (%)			0.95			0.42		< 0.001
Normal	115 (44.2)	538 (44.4)		432 (36.6)	218 (39.9)		1572 (27.2)	
Overweight	74 (28.5)	334 (27.6)		387 (32.8)	172 (31.5)		1898 (32.8)	
Obese	71 (27.3)	340 (28.1)		360 (30.5)	156 (28.6)		2318 (40.1)	
Smoking status 6 months prior to diagnosis, n (%)			0.44			0.04		0.09
Never smoker	160 (61.5)	772 (63.7)		605 (51.3)	316 (57.9)		3020 (52.2)	
Current smoker	33 (12.7)	119 (9.8)		93 (7.9)	31 (5.7)		465 (8.0)	
Former smoker	43 (16.5)	224 (18.5)		372 (31.6)	161 (29.5)		1892 (32.7)	
Unknown	24 (9.2)	97 (8.0)		109 (9.3)	38 (7.0)		411 (7.1)	
Had diabetes at diagnosis, n (%)	12 (4.6)	57 (4.7)	0.95	218 (18.5)	71 (13.0)	0.005	1018 (17.6)	0.46
Had dyslipidemia at diagnosis, n (%)	40 (15.4)	145 (12.0)	0.13	615 (52.2)	252 (46.2)	0.02	3165 (54.7)	0.11
Had hypertension at diagnosis, n (%)	30 (11.5)	164 (13.5)	0.39	672 (57.0)	261 (47.8)	< 0.001	3224 (55.7)	0.41
Had prevalent CVD at diagnosis, n (%)	2 (0.8)	3 (0.3)	0.19	75 (6.4)	18 (3.3)	0.009	228 (3.9)	< 0.001

1 Based on studentized T-test for continuous variables and Chi-square test for categorical variables.

Abbreviations: AJCC, American Joint Committee on Cancer; CVD, cardiovascular disease; GED, general education degree; SD, standard deviation.

**Table 2:** Hazard rate ratios and 95% confidence intervals of incident cardiometabolic risk factors women with hormone-receptor positive breast cancer with history of endocrine therapy use relative to non-endocrine therapy use

	Premenopausal	Postmenopausal			
*Cardiometabolic Risk Factor*	Non-users of Endocrine Therapy, n = 260	Tamoxifen Users, n = 1,212	Non-users of Endocrine Therapy, n = 1,179	Tamoxifen Users, n = 546	Aromatase Inhibitor Users, n = 5,788
Diabetes					
Total events	10	95	64	36	533
Total Person-Years	2,255.5	9,788.7	6,944.0	3,595.0	33,370.6
Incidence Rate per 1,000 person-years	4.43	9.71	9.22	10.01	15.97
Unadjusted model, HR (95%CI)	Referent	2.15 (1.12, 4.12)	Referent	1.15 (0.77, 1.73)	1.74 (1.34, 2.25)
Adjusted model^1^, HR (95%CI)	Referent	1.65 (0.83, 3.25)	Referent	1.22 (0.79, 1.89)	1.37 (1.05, 1.80)
Dyslipidemia					
Total events	26	150	121	60	846
Total Person-Years	1,879.1	8,667.9	3,537.0	1,903.8	13,630.6
Incidence Rate per 1,000 person-years	13.84	17.31	34.21	31.52	62.07
Unadjusted model, HR (95%CI)	Referent	1.24 (0.81, 1.87)	Referent	0.96 (0.71, 1.31)	1.62 (1.34, 1.96)
Adjusted model^1^, HR (95%CI)	Referent	1.23 (0.80, 1.90)	Referent	0.92 (0.67, 1.27)	1.58 (1.29, 1.92)
Hypertension					
Total events	34	160	132	81	874
Total Person-Years	1,934.8	8,376.4	2,897.9	1,681.2	13,123.6
Incidence Rate per 1,000 person-years	17.57	19.10	45.55	48.18	66.60
Unadjusted model, HR (95%CI)	Referent	1.07 (0.74, 1.55)	Referent	1.13 (0.86, 1.49)	1.39 (1.16, 1.67)
Adjusted model^1^, HR (95%CI)	Referent	1.07 (0.72, 1.58)	Referent	1.43 (1.06, 1.92)	1.50 (1.24, 1.82)

1 Adjusted for age, race/ethnicity, smoking status, AJCC stage, body mass index, chemotherapy, radiation therapy, breast surgery, and prevalent (baseline) diabetes, dyslipidemia, and hypertension (when not the outcome of interest).

**Table 3:** Adjusted^1^ hazard rate ratios and 95% confidence intervals of incident cardiometabolic risk factors in women with hormone-receptor positive breast cancer by endocrine therapy use stratified by baseline body mass index groups

	Premenopausal Tamoxifen Users	p-value for interaction	Postmenopausal Tamoxifen Users	p-value for interaction	Aromatase Inhibitor Users	p-value for interaction
	HR (95% CI)		HR (95% CI)		HR (95% CI)	
Normal Weight						
Diabetes	Not Estimatable		0.76 (0.26, 2.20)		1.25 (0.69, 2.27)	
Dyslipidemia	1.07 (0.49, 2.35)		0.82 (0.47, 1.45)		1.71 (1.22, 2.38)	
Hypertension	0.76 (0.37, 1.56)		1.57 (0.97, 2.56)		1.45 (1.03, 2.04)	
Overweight						
Diabetes	3.09 (0.66, 14.50)	NA	0.52 (0.22, 1.24)	0.83	1.19 (0.76, 1.85)	0.98
Dyslipidemia	0.81 (0.40, 1.63)	0.37	1.11 (0.61, 2.02)	0.30	1.84 (1.27, 2.68)	0.77
Hypertension	1.55 (0.75, 3.23)	0.43	1.19 (0.68, 2.08)	0.60	1.57 (1.13, 2.19)	0.72
Obese						
Diabetes	1.07 (0.48, 2.39)	NA	2.05 (1.13, 3.74)	0.08	1.49 (0.98, 2.26)	0.54
Dyslipidemia	2.33 (0.96, 5.69)	0.38	0.84 (0.48, 1.47)	0.95	1.24 (0.89, 1.72)	0.14
Hypertension	1.21 (0.64, 2.27)	0.38	1.40 (0.83, 2.35)	0.90	1.46 (1.05, 2.03)	0.73

1 Adjusted for age, race/ethnicity, smoking status, AJCC stage, chemotherapy, radiation therapy, breast surgery, and prevalent (baseline) diabetes, dyslipidemia, and hypertension (when not the outcome of interest).

## Data Availability

These data are not publicly available because they contain potentially identi able information but may be available from Kaiser Permanente Northern California contingent on appropriate human subjects’ approval and necessary data use agreements.

## Abbreviations

AJCC: American Joint Committee on Cancer
AI: aromatase inhibitor
BC: breast cancer
BMI: body mass index
CVD: cardiovascular disease
CI: confidence interval
EHR: electronic health record
GED: general education degree
HR: hazard rate ratio
ICD: International Classification of Disease
KPNC: Kaiser Permanente Northern California
SD: standard deviation
